# Analysis of cutaneous leishmaniasis among military personnel in the Islamic Republic of Iran: a spatiotemporal study between 2018 and 2022, trend forecasting based on ARIMA model

**DOI:** 10.1186/s12879-024-10200-x

**Published:** 2024-11-16

**Authors:** Reza Tadayonfar, Arasb Dabbagh-Moghaddam, Mohammad Barati, Mohammad Hassan Kazemi-Galougahi, Zahra Aminifarsani, Nahid Jalallou, Mohammad Reza Shirzadi, Faranak Ghrachorloo, Ramin Khaghani

**Affiliations:** 1 Infectious Diseases Research Center, AJA University of Medical Sciences, Tehran, Iran; 2 Department of Preventive Medicine, AJA University of Medical Sciences, Tehran, Iran; 3 Department of Social Medicine, Faculty of Medicine, AJA University of Medical Sciences, Tehran, Iran; 4 Bayesian Imaging and Spatial Statistics Group, Institute for Statistics, Ludwig-Maximilians University of Munich, Munich, Germany; 5 Department of Laboratory Science, School of Paramedicine, AJA University of Medical Sciences, Tehran, Iran; 6 Center for Communicable Diseases Control, Ministry of Health and Medical Education, Tehran, Iran; 7 Department of Military Health, Faculty of Medicine, AJA University of Medical Sciences, Tehran, Iran

**Keywords:** Cutaneous leishmaniasis, Military personnel, Spatio-temporal analysis, ARIMA, Iran

## Abstract

**Background:**

Cutaneous leishmaniasis is one of the few infectious diseases whose global prevalence is on the rise. Iran ranks among the eight most affected countries in the world. Iranian military personnel are often sent to endemic areas for cutaneous leishmaniasis without prior immunity, and they have fewer health facilities in military centers than the general population. This study aims to comprehensively investigate the situation of cutaneous leishmaniasis in Iranian military personnel across all units from 2018 to 2022 and predict the disease trend using time series analysis up to the end of 2025.

**Methods:**

We analyzed data from the Iranian Ministry of Health to perform spatiotemporal and descriptive analyses based on patient frequency. Variables examined included age distribution, cutaneous leishmaniasis types (zoonotic or anthroponotic), month of healthcare facility visits, and lesion locations. This study employed the ARIMA model (*p* = 2, d = 0, q = 1)(*P* = 3, D = 0, Q = 0), for time series analysis and forecasting the disease trend up to 36 months after 2022.

**Results:**

Over five years, 2,894 patients were reported. The Esfahan, Khuzestan, and Ilam provinces had the highest average patient counts, with hot spots primarily found in central, south, southwestern, and western Iran. Although the total number of patients with zoonotic cutaneous leishmaniasis was almost equal to anthroponotic cutaneous leishmaniasis, in high-risk provinces such as Esfahan, Khuzestan, and Ilam, the confirmed cases of zoonotic cutaneous leishmaniasis were much more than anthroponotic cutaneous leishmaniasis. patient numbers peak in October and November. Demographic analysis revealed that younger patients outnumbered older patients. Lesion locations were frequent on the forelimbs and lower limbs. The time series analysis for 36 months after 2022 indicated the seasonal pattern of the disease and predicted an upward trend after 2022.

**Conclusion:**

While overall cases have declined, provinces such as Esfahan exhibit an upward trend. The expansion of hotspots from the west and southwestern to the center and south of Iran, coupled with an increasing trend in time series analysis, suggests the potential emergence of new foci and a rise in patient numbers in the future. In provinces with high disease prevalence, preventive measures should be prioritized, particularly in Ilam, Khuzestan, and Esfahan.

**Supplementary Information:**

The online version contains supplementary material available at 10.1186/s12879-024-10200-x.

## Background

Cutaneous leishmaniasis (CL) is a prevalent type of leishmaniasis transmitted by species of sandflies [1]. CL is among the few infectious diseases whose global prevalence is increasing, largely due to warfare and environmental influences, particularly in the Middle East and the Americas, where it is most common [2]. The World Health Organization (WHO) reported that in 2018 over 85% of newly identified CL cases were traced back to Afghanistan, Algeria, Bolivia, Brazil, Colombia, Iran, Iraq, Pakistan, Syria, and Tunisia [3]. Iran has officially reported between 15,000 and 20,000 cases of CL annually and is one of the eight most affected countries. In Iran, CL is endemic in two forms: zoonotic CL (ZCL) caused by *Leishmania major* and anthroponotic CL (ACL) caused by *Leishmania tropica* [4]. The primary reservoirs for *Leishmania* in Iran include *Rhombomys optimus*,* Meriones libycus*, and *Tatera indica.* Phlebotomus sandflies are the principal vectors of CL in Iran, with *Phlebotomus papatasi* being the main vector for *Leishmania major* across all ZCL areas. Other potential vectors include *Phlebotomus salehi*,* Phlebotomus ansarii*,* Phlebotomus caucasicus*,* Phlebotomus mongoliensis*,* Phlebotomus andrejevi*,* Phlebotomus alexandri*, and *Phlebotomus bergeroti.* Additionally, *Phlebotomus sergenti* is responsible for *ACL* [5].

CL involves a complex ecological cycle influenced by geographical location and climate [6–9]. Geographic Information Systems (GIS) play a crucial role in assessing the impact of these factors on disease incidence. They help analyze disease geography and health outcomes over time [10]. In addition to spatiotemporal analysis by GIS, Time-series analysis, based on historical data, aids in developing hypotheses about disease dynamics using extrapolated models [11, 12]. The autoregressive integrated moving average (ARIMA) model is widely used in medical research for practical time-series analysis [13–15].

Frequently lacking prior immunity, Iranian military personnel (IMP) are commonly sent to regions where the disease is endemic for either training or missions [16, 17]. Additionally, in the military population, factors such as limited access to bathrooms, poor hygiene among young soldiers, particularly those with low literacy, exposure to soil and contaminated water during training exercises, and other similar conditions increase the risk of skin diseases, including CL [18, 19].

Numerous research articles have focused on leishmaniasis’s epidemiological and geographical trends within Iran’s general population (IGP) [20, 21]. However, few comprehensive studies exist on how CL is distributed among specific groups, such as IMP [8, 22]. Additionally, no studies have been carried out to predict the trend of leishmaniasis in IMP. The status of this disease among IMP has not been thoroughly studied since 2018. This study aims to comprehensively investigate the situation of CL in IMP across all units from 2018 to 2022 and predict the disease trend using time series analysis up to the end of 2025.

## Methods

### Study regions

Iran is the second-largest country in the Middle East, covering an area of 1,648,195 square kilometers. It shares borders with the Caspian Sea, the Persian Gulf, and the Gulf of Oman and is surrounded by Afghanistan, Armenia, Azerbaijan, Iraq, Pakistan, Turkey, and Turkmenistan. The country boasts diverse landscapes, ranging from the Caspian Sea at -28 m to Mount Damavand at 5,610 m, resulting in varied climates and environmental conditions. Temperature fluctuations are extreme, with recorded temperatures ranging from below − 30 °C to above 40 °C and varying rainfall averages across the country [23].

### Study design and data collection

Data related to CL from 2018 to 2022 were registered with the Ministry of Health and Medical Education of Iran. The research analyzed several variables, including geographic location of employment, demographic age distribution, month of disease diagnosis, type of CL (ACL or ZCL), and anatomical sites of lesions. This study employed time series analysis to forecast the disease trend for up to 36 months following 2022.

### Diagnostic methods

In Iran’s medical centers, the CL diagnostic protocol begins with evaluating clinical signs. The following steps involve considering regional epidemiological data. Following this initial evaluation, a biopsy is performed, and Giemsa staining is used to identify Leishman’s bodies, thereby confirming the diagnosis of CL.

### Statistical analysis

Descriptive statistical analysis was conducted using Microsoft Excel 2013. The Cochran-Armitage linear trend test was employed to evaluate trends and changes in patient frequency over time using STATA version 11.

### Spatial analysis

For spatial analysis, we utilized ArcGIS version 10.3. The foundational data for these analyses included CL frequency across various provinces in Iran. This methodology enabled us to detect high-risk provinces, hotspots, clusters, and outliers throughout the country.

Choropleth maps were used to visualize changes in the distribution of CL cases over the study period, highlighting high-risk provinces through color variations.

Hotspot analysis identifies statistically significant spatial clusters of high values (hot spots) and low values (cold spots). It generates an output with a z-score, p-value, and confidence level bin field (Gi_Bin) for each feature in the input dataset. Based on these parameters, a map is drawn. The z-scores and p-values serve as measures of statistical significance that indicate whether or not to reject the null hypothesis (random distribution). They effectively reveal whether the observed spatial clustering of high or low values is more pronounced than expected in a random distribution of those same values.

A high z-score and small p-value for a feature indicate a spatial clustering of high values, while a low negative z-score and small p-value suggest a spatial clustering of low values. The higher (or lower) the z-score, the more intense the clustering. Conversely, a z-score near zero indicates no apparent spatial clustering. The Gi_Bin field identifies statistically significant hot and cold spots: features within ± 3 bins indicate a 99% confidence interval (CI), those within ± 2 bins indicate a 95% CI, and those within ± 1 bins indicate a 90% CI. Nonsignificant clustering is represented in bin 0. In this analysis, the identification of hot and cold spots is not solely determined by the prevalence of patients in a given area; instead, the proximity of an area to neighboring regions and the status of adjacent zones also play crucial roles in defining its condition. Specifically, even if an area does not exhibit a high patient count, it may still be classified as a hot spot if high-prevalence areas surround it [24, 25].

Additionally, the Anselin Local Moran’s I method was applied for a more nuanced identification of clusters and outliers, enhancing the hotspot analysis. This tool evaluates spatial clusters and outliers based on a specified set of features and an analysis field. It calculates a local Moran’s I value, z-score, pseudo-p-value, and cluster type code for each statistically significant feature. A positive Moran’s I indicate that a feature is part of a cluster with neighboring features exhibiting similar high or low values. In contrast, a negative value signifies an outlier surrounded by dissimilar values. For significance, the p-value must be sufficiently low; typically, values below 0.05 are considered significant. This method differentiates between high-high clusters (high-risk areas surrounded by similar regions), high-low outliers (high-risk areas adjacent to low-risk areas), low-high outliers (low-risk areas adjacent to high-risk areas), and low-low clusters (low-risk areas surrounded by similar regions).

About hotspot analysis and Anselin Local Moran’s I correlation, hotspot analysis focuses on identifying clusters of high and low values. At the same time, Anselin Local Moran’s I emphasizes evaluating spatial correlation and local patterns. Hotspot analysis typically results in maps of hot and cold spots, whereas Anselin Local Moran’s I provides insights into spatial relationships and outliers. In practice, these methods can complement each other in spatial analysis projects, offering more comprehensive views of the data [24, 26].

### Forecasting

The ARIMA model was created to leverage the relationships in sequentially lagged data collected periodically [27]. The autoregressive (AR) component defines the output variable as linearly dependent on its prior values. Concurrently, the moving average (MA) represents the linear regression of the current value of the series against both the current and previous values. If the raw data indicates signs of nonstationarity, a differencing step (the integrated component of the model) should be used to eliminate it [28].

The ARIMA model consists of three parameters: p, d, and q, which correspond to the order of the autoregressive, integrated, and moving average components, respectively. The parameter P represents the order of seasonal autoregression; D indicates the extent of seasonal differencing; and Q denotes the order of seasonal moving average. Given that the data exhibited signs of seasonal behavior, the general multiplicative seasonal model [ARIMA (p, d,q)×(P, D, Q)s)] was applied [29].

Evaluating diagnostic parameters was performed using the Akaike Information Criterion (AIC), Bayesian Information Criterion (BIC), and log-likelihood to assess the models’ goodness-of-fit and determine statistical significance (*P* < 0.05). lower values for AIC and BIC and higher values for log-likelihood are preferred. Considering all three metrics together ensures a more robust evaluation of model performance. The Ljung–Box test was utilized to verify if the sequence of residual errors displayed white noise characteristics [30, 31]. Ultimately, predictive analysis could be executed using the optimal combination of parameters. All analyses were carried out with Minitab - version 17.

## Results

**Fig. 1:**
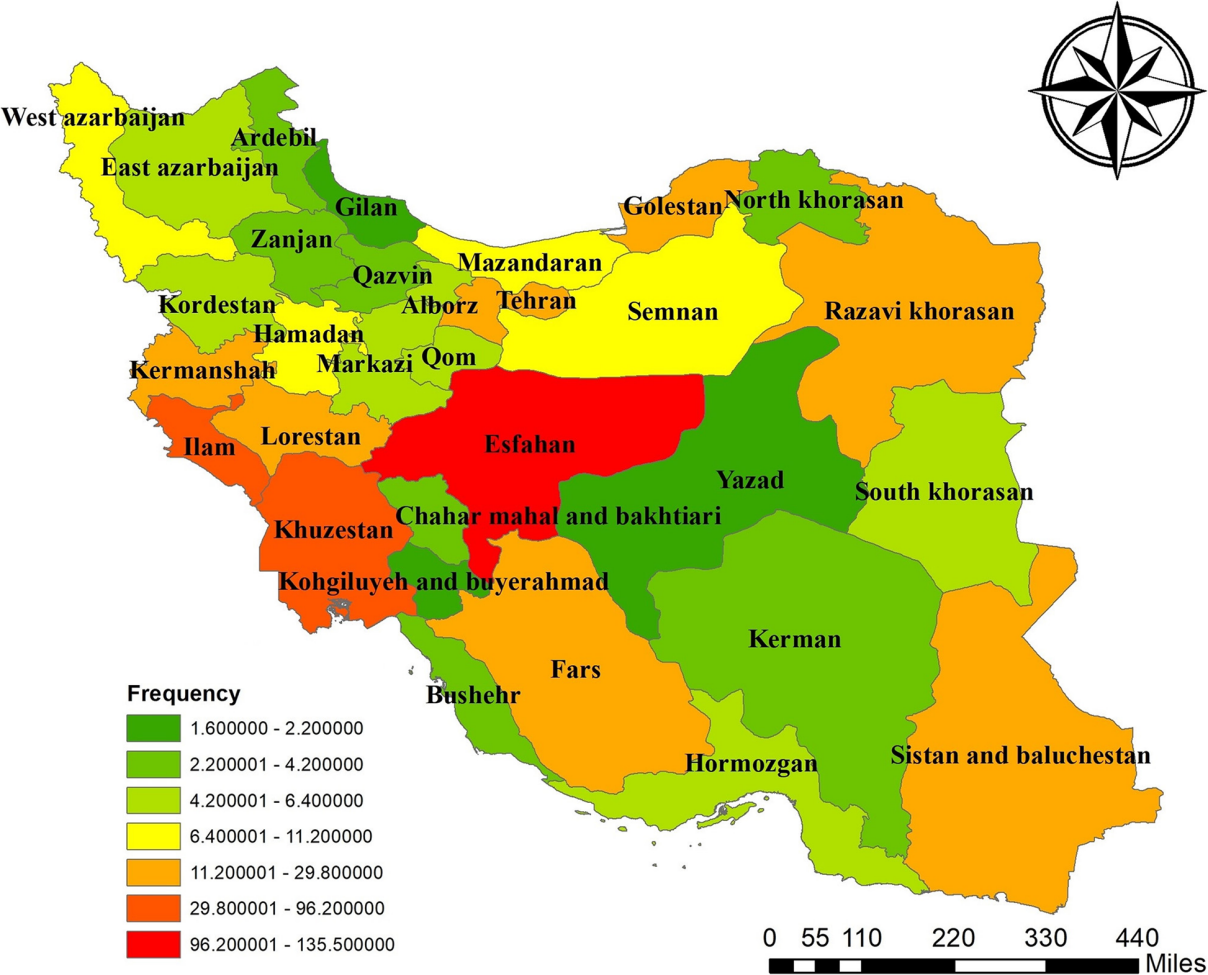
Choropleth map depicting each province’s average number of CL patients over five years

Recent data revealed 2,894 cases of CL in IMP patients from 2018 to 2022. The Cochran-Armitage trend test analysis indicated a statistically significant linear decline in annual patient counts (*p* < 0.001). Over these five years, the number of patients decreased by 19.01%. The distribution of cases across provinces is detailed in Table 1. A choropleth map (Fig. 1) also illustrates the average patient frequency per province during the study period, highlighting Esfahan, Khuzestan, and Ilam as high-risk areas.

**Table 1:**
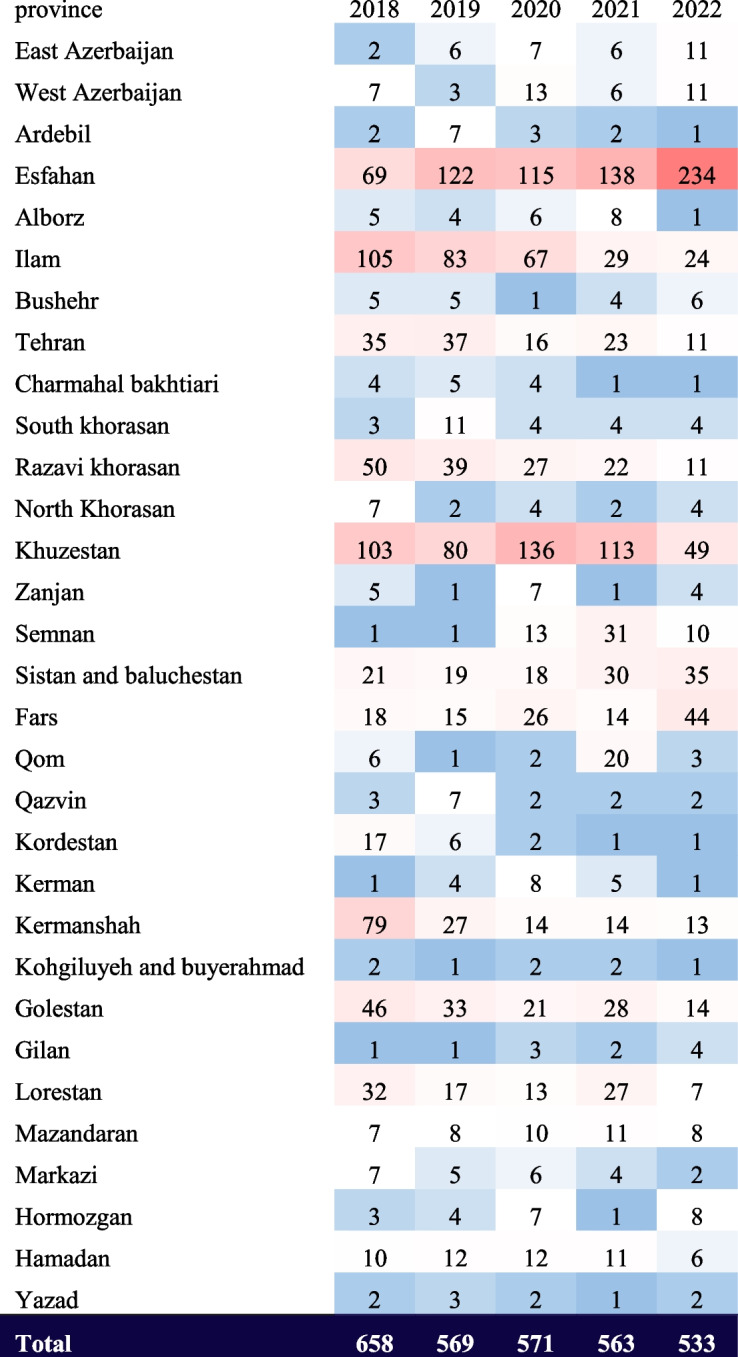
Annual patient numbers for each province

The spectrum ranges from blue (indicating low patient numbers) to red (indicating high patient numbers)

**Fig. 2:**
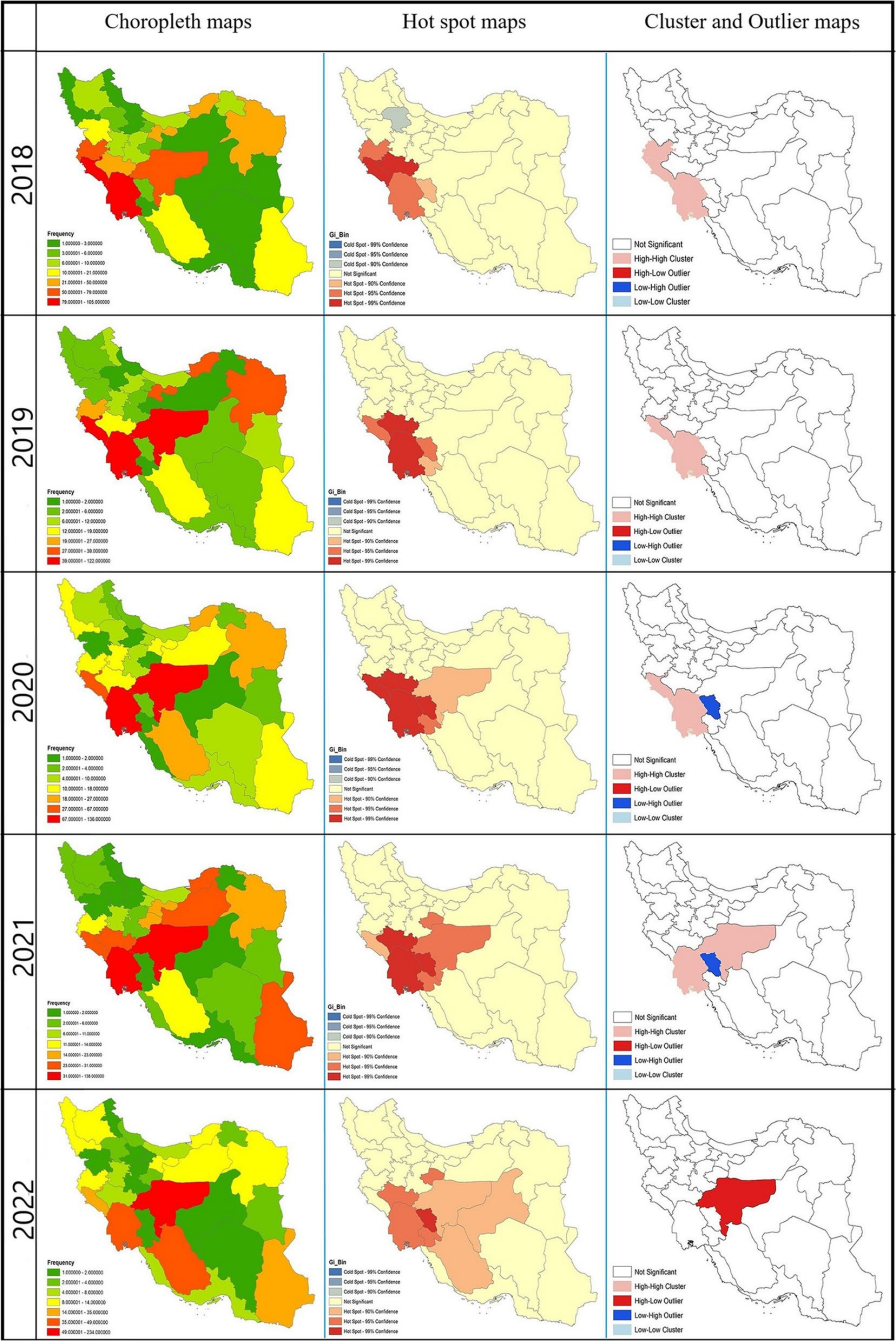
Maps displaying the spatial distribution of CL in IMP from 2018 to 2022.The left column displays annual maps of CL, illustrating the number of patients using a color gradient that ranges from green (indicating low risk) to red (indicating high risk). The middle column focuses on hotspot analysis with annual maps, where red represents hotspots, blue denotes cold spots, and cream indicates areas that are neither classified as hot nor cold. Lastly, the right column presents annual maps based on Anselin Local Moran’s I analysis, where clusters and outliers are distinguished by different colors depending on their type

The choropleth maps showing patient frequency from 2018 to 2022 are presented In the left column of Fig. 2. Among the provinces identified as high-risk in 2018, the number of patients decreased in all these provinces except for Esfahan in subsequent years.

The maps related to hotspot analysis are presented in the middle column of Fig. 2 with detailed information. 2018, five hotspots (Ilam, Lorestan, Kermanshah, Khuzestan, Charmahal and Bakhtiari) and one cold spot (Zanjan) were identified. In subsequent years, Esfahan, Kohgiluyeh and Buyer Ahmad, Qom, and Fars were also recognized as hotspots.

The analysis conducted via Anselin Local Moran’s I illustrated on the right side of Fig. 2, revealed insightful spatial clustering trends between 2018 and 2022. In 2018, this analysis identified the provinces of Kermanshah, Ilam, and Khuzestan as high-high clusters. In the following years, with increased disease cases in Esfahan, this province was recognized as a high-high cluster alongside Iran’s western and southwestern provinces. Provinces with low frequency of the CL cases, such as Charmahal and Bakhtiari, which were situated between high-high clusters, were classified as low-high outlier. By the end of the study period, due to an increase in patient numbers in Esfahan and a decrease in neighboring provinces, Esfahan was identified as a high-low outlier.

**Fig. 3:**
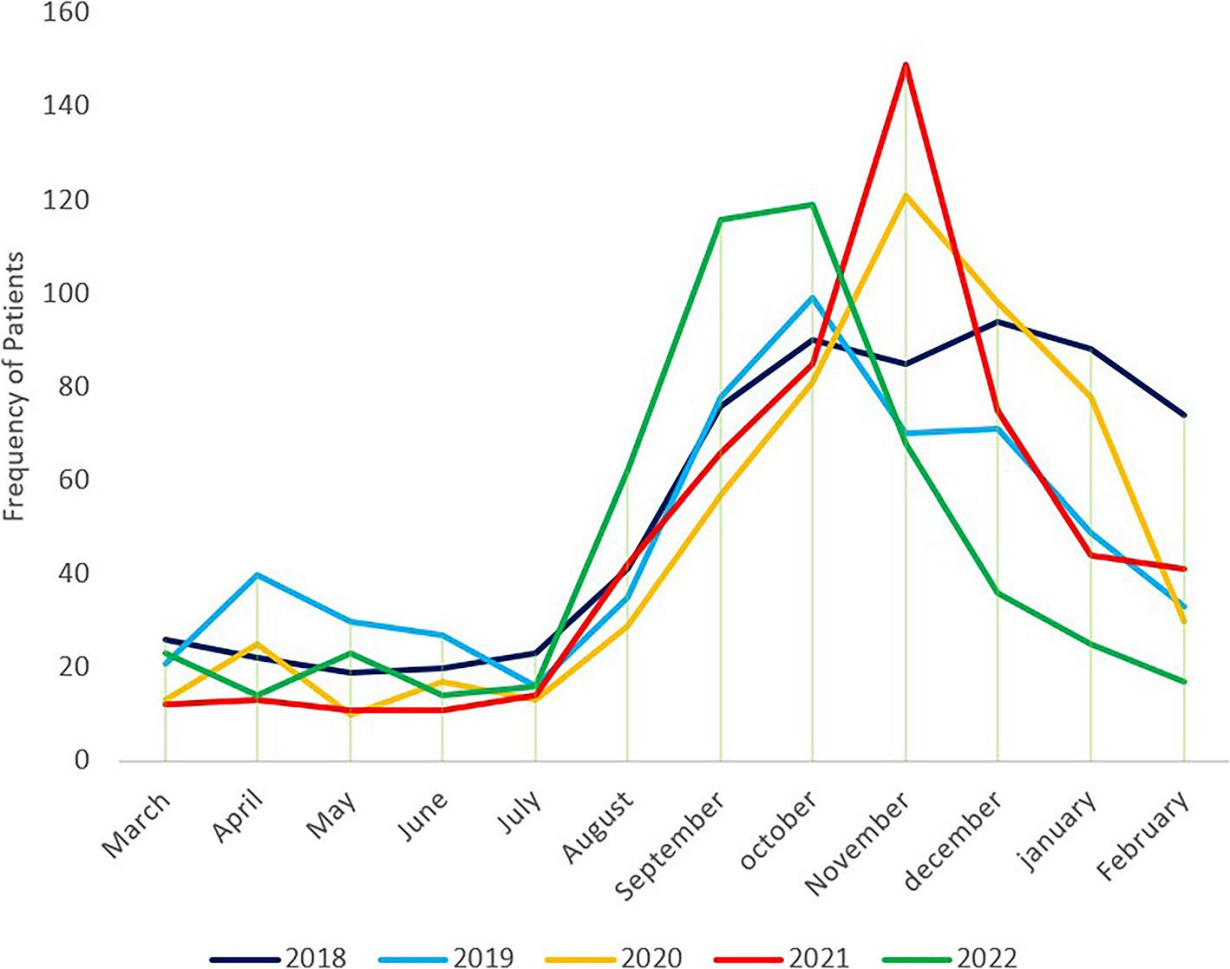
The monthly distribution of CL patients for each year

Paraclinical findings revealed that 26% of patients were diagnosed with ZCL. In comparison, 23% were diagnosed with ACL, with the specific type of leishmaniasis undetermined in 51% of patients. Notably, ZCL was more prevalent than ACL in high-risk provinces, particularly in Esfahan, Khuzestan, and Ilam (Table 2). Monthly temporal analysis indicated increased CL cases beginning in July and August, peaking in October and November, as demonstrated in Fig. 3. Demographic analysis showed that 70.8% of patients were between 18 and 30, and 29.7% were between 31 and 65. Clinical examination data revealed that 83.2% of patients presented with cutaneous lesions on the forelimbs and lower limbs. Among these, 6.71% exhibited lesions on the trunk, 5.36% on the face, and 4.71% on the head and neck.

**Table 2:** Frequency of ACL and ZCL in different provinces of Iran from 2018 to 2022

Province	ZCL	ACL	No data
**East Azerbaijan**	1	0	31
**West Azerbaijan**	2	4	34
**Ardebil**	0	0	33
**Esfahan**	389	18	274
**Alborz**	0	2	25
**Ilam**	82	5	223
**Bushehr**	0	1	20
**Tehran**	6	4	113
**Charmahal and bakhtiari**	1	1	13
**South Khorasan**	0	10	16
**Razavi Khorasan**	29	47	73
**North Khorasan**	4	2	13
**Khuzestan**	346	68	62
**Semnan**	21	2	18
**Sistan and baluchestan**	46	5	72
**Fars**	30	11	72
**Qazvin**	2	3	8
**Qom**	1	0	33
**Kordestan**	3	1	23
**Kerman**	3	13	3
**Kermanshah**	14	9	124
**Kohgiluyeh and buyerahmad**	0	0	8
**Golestan**	85	6	51
**Gilan**	3	1	7
**Lorestan**	6	5	85
**Mazandaran**	10	19	15
**Markazi**	1	3	20
**Hormozgan**	1	3	19
**Hamedan**	1	0	50
**Yazd**	6	0	4

There was no para-clinic data for many patients to determine the type of leishmaniasis

**Fig. 4:**
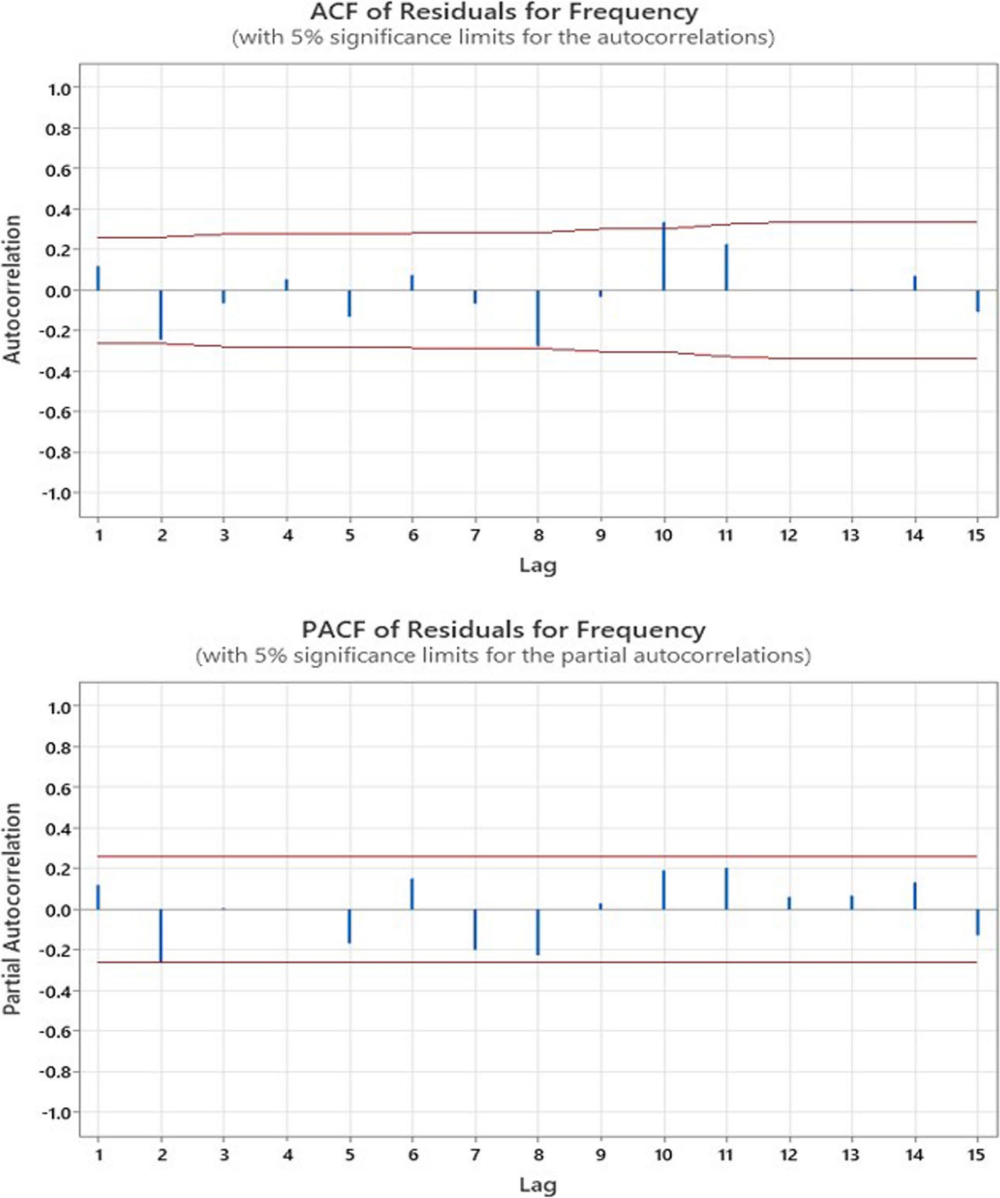
ACF and PACF plots

For time series analysis, the Autocorrelation Function (ACF) and Partial Autocorrelation Function (PACF) are used to determine model specification (Fig. 4). ACF measures the correlation between a time series and its lagged values, helping to identify seasonality and determine the order of the MA component in ARIMA models. PACF, on the other hand, measures this correlation while controlling for shorter lags, providing a clearer understanding of the direct relationship between current observations and their lags. It is beneficial for identifying the order of the AR components in ARIMA models. The “lag” in ACF and PACF plots represents the number of time periods by which the observations are shifted. For example, Lag 1 refers to the correlation between observations that are one time period apart. Lag 2 refers to the correlation between observations that are two time periods apart, and so on. Each lag value indicates how far back in time we are looking to find correlations between current and past observations. The ACF and PACF plots in Fig. 4 indicate Oscillation in lag values; some lags show positive correlations, and others show negative correlations. This can indicate several things, such as Seasonality or Cyclical Patterns. Alternating positive and negative values may suggest the data’s underlying seasonal or cyclical patterns. For example, a positive correlation at one lag followed by a negative correlation at the next may indicate that the influence of past values reverses after a certain period. Based on this result, the seasonal autoregressive integrated moving average (SARIMA) is a suitable model for this study.

This study employed the SARIMA model, specifically ARIMA (*p* = 2, d = 0, q = 1)(*P* = 3, D = 0, Q = 0), for time series analysis and forecasting. The model was selected based on the lowest Akaike Information Criterion (AIC) and Bayesian Information Criterion (BIC) values. An alternative model with parameters d = 0, D = 0, *p* = 2, q = 1, *P* = 3, and Q = 0 was also considered. The selection criteria yielded a log-likelihood of 13319.8, resulting in AIC_c = -26623.4, AIC = -26625.6, and BIC = -26610.9 ( see additional file 1).

The estimated parameters for the selected model are summarized in Table 3. The autoregressive coefficients (AR) for lags 1 and 2 are statistically significant, with values of 1.444 (*p* < 0.001) and − 0.447 (*p* = 0.003), respectively. Significant seasonal autoregressive coefficients (SAR) for lags 12, 24, and 36 indicate strong seasonal patterns in the data (*p* < 0.001). The moving average coefficient (MA) for lag 1 is also significant at *p* < 0.001, with a value of 1.0193.

**Table 3:** Final estimates of parameters

Type	Coef	SE Coef	T-Value	*P*-Value
AR 1	1.444	0.138	10.45	0.000
AR 2	-0.447	0.141	-3.17	0.003
SAR 12	0.457	0.101	4.53	0.000
SAR 24	-0.465	0.125	-3.70	0.001
SAR 36	1.002	0.108	9.25	0.000
MA 1	1.0193	0.0114	89.44	0.000

To assess model adequacy, we conducted the Modified Box-Pierce test (Ljung-Box) for autocorrelation of residuals at various lags (12, 24, 36, and 48) (Table 4). Results indicate that while the Chi-Square statistic for lag 12 was statistically significant (*p* < 0.001), indicating potential autocorrelation in the residuals, subsequent lags showed p-values suggesting that residuals were adequately modeled: *p* = 0.086 for lag 24, *p* = 0.615 for lag 36, and *p* = 0.958 for lag 48. The Ljung-Box test indicated that the model is generally well-fitted, especially in high lags.

**Table 4:** Modified Box-Pierce (Ljung-Box) Chi-Square Statistic

Lag	12	24	36	48
Chi-Square	24.85	26.64	27.16	27.59
DF	6	18	30	42
P-Value	0.000	0.086	0.615	0.958
Lag	12	24	36	48
Chi-Square	24.85	26.64	27.16	27.59

**Fig. 5:**
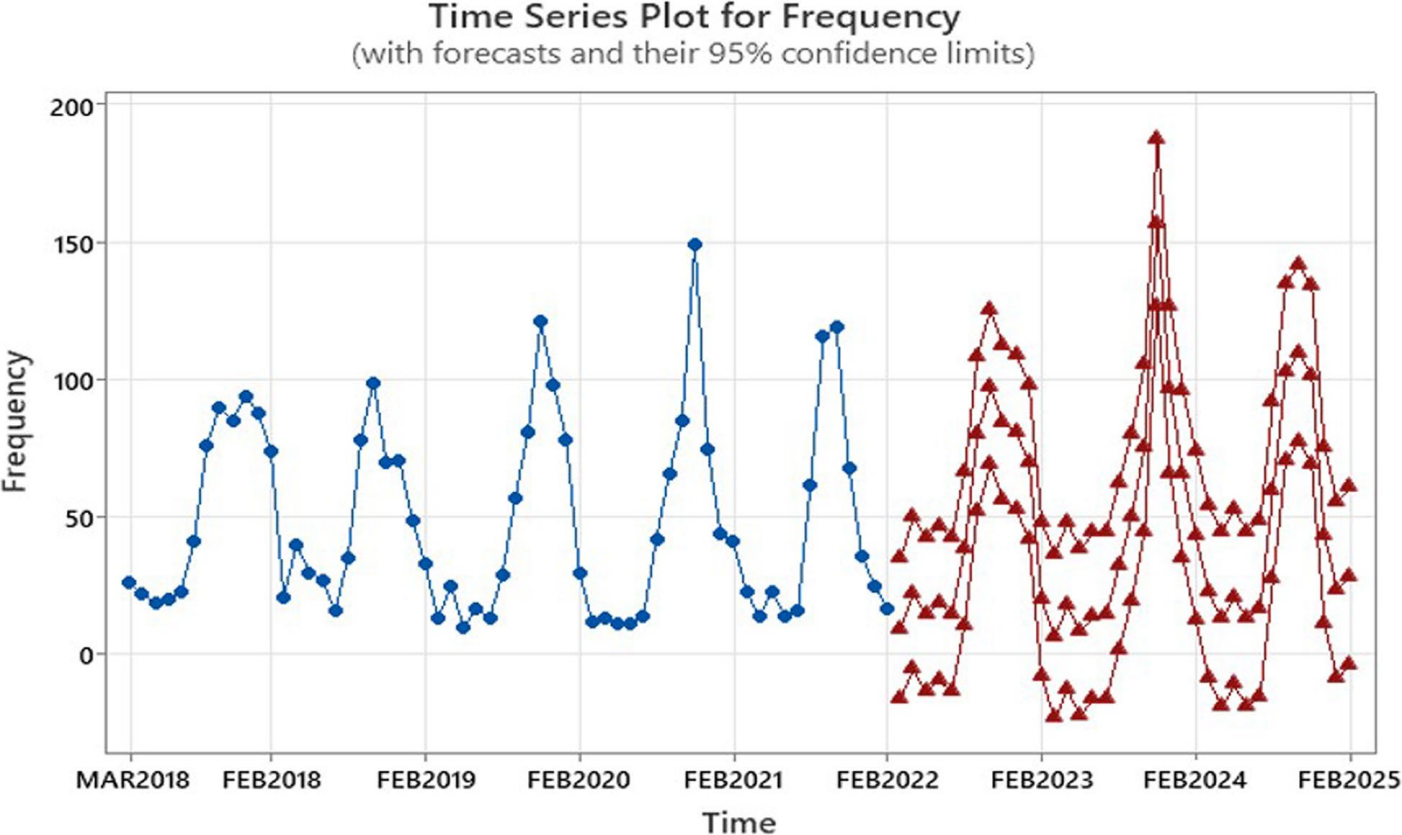
Time series plot for frequency

The forecast results for the 36 months following 2022, presented in Fig. 5, The forecasts are accompanied by 95% confidence intervals, which indicate the range of values within which the actual number of patients Is likely to be found. The results indicate that the number of patients follows a seasonal pattern. This aligns with the seasonal trends observed in leishmaniasis cases from 2018 to 2022, characterized by increased patient numbers from July and Agust to October and November. The 95% confidence intervals for the forecasts are wide, indicating a high degree of uncertainty regarding the future number of leishmaniasis patients. However, the overall trend is clear: the number of patients is expected to increase over the next 36 months (See additional file 2).

## Discussion

Over five years, from 2018 to 2022, the annual number of patients among the IMP showed a linear decrease. This observation is corroborated by evidence indicating a decline in CL incidence and prevalence rates among IGP from 1983 to 2020 [20, 21]. These findings contrast with previous studies on CL in IMP patients, which reported nonlinear fluctuations in patient counts from 2005 to 2014, characterized by periods of both increase and decrease [22].

From 2018 to 2019, hotspots and high-high clusters for CL were predominantly observed in the western and southwestern regions of Iran. However, the following years noted a subsequent expansion toward the country’s central and southern parts. Over these five years, Ilam, Khuzestan, and Esfahan were identified as high-risk provinces, although Ilam and Khuzestan exhibited a declining trend. In alignment with our findings, investigations into CL among IMP from 2005 to 2017 underscored Khuzestan and Esfahan as the hyper-endemic provinces of CL [8, 22, 32]. Moreover, extensive investigations concerning CL in IGP from 1983 to 2020 have effectively identified noteworthy clusters of high-risk areas in the country’s western, south, southwestern, and central regions [20, 21, 33].

Iran’s colder, high-altitude provinces are typically unsuitable for the spread of CL and its vectors [34]. However, this study indicates that despite having few patients and an unsuitable environment, Lorestan, Charmahal Bakhtiari, and Kohgiluyeh and BuyerAhmad emerged as annual CL hotspots. This phenomenon can be attributed to their proximity to Khuzestan and Esfahan, which are recognized as high-risk provinces. Anselin Local Moran’s I analysis supports this idea by not indicating that these provinces are high-high clusters. Yazd Province was identified as a hotspot in 2022, despite the lack of significant change in the annual patient count. This classification appears to be influenced by Yazd’s proximity to Esfahan and Fars provinces, which reported an increase in patient numbers in 2022. While there is no prior data on CL among Yazd military personnel, existing research on the IGP has identified Yazd as an endemic area for CL. The difference in patient frequency among the Yazd general and military population is probably due to the few military centers in this province [20–22, 32].

Although the total number of identified ZCL and ACL cases was similar over the five years, in high-risk provinces such as Esfahan, Khuzestan, and Ilam, ZCL cases were higher than ACL. The prevalence of ZCL among military personnel is expected, as military bases are typically located outside urban areas, leading to increased contact with reservoirs and vectors. ZCL is widespread in various regions of Iran, including Esfahan, Khuzestan, and Ilam. The sand fly species Phlebotomus papatasi serves as a significant vector for ZCL, while reservoir hosts such as Rhombomys Optimus, Meriones hibiscus, and Tatera indica facilitate its spread across the country [35].

A comparison of patient frequency each month revealed an increase from July or August to October or November, correlating with sandfly activity and the Leishmania incubation period. CL lesions peak during this time, coinciding with the monsoon season [36]. In Iran, most cases are detected from September to December, when temperatures range from 23 °C to 27 °C, creating ideal conditions for sandfly reproduction [9]. In Esfahan Province, CL incidence is strongly related to temperature, with sandflies being more active during dry or moderate seasons, such as late summer and early winter [37]. In western Iran, CL incidence peaks in winter, particularly in January and February, following the disease’s incubation period [38]. Warmer temperatures accelerate the maturation of the Leishmania parasite, increasing the risk of infection. Sufficient moisture is also essential for sandfly growth, as humidity aids egg survival. However, heavy rainfall can decrease sandfly populations by reducing suitable resting sites and limiting flight activity [37, 39–41]. Increased humidity and warm days in the southwest significantly contribute to CL incidence [42]. Conversely, heavy rainfall and flooding in northern and northwestern Iran reduce the number of sandfly breeding sites, decreasing CL cases [43].

Our data indicate that there are more young patients than older individuals, likely due to their increased involvement in military operations and maneuvers, heightening their exposure to disease vectors. The prevalence of Leishmania infection among older military personnel may be higher than reported figures suggest. This could be due to prior exposure to the pathogen, which may have conferred immunity, making subsequent infections asymptomatic. Our findings are consistent with previous research on CL in the IGP, which also reported a higher number of younger patients than older ones [20].

Consistent with our research, other investigations on CL in IGP revealed that most patients presented with forelimbs and lower limbs lesions [20, 44]. Since these body parts are frequently uncovered, they are more susceptible to insect bites, resulting in more lesions.

Several studies have used ARIMA models to fit and predict the changing trends of infectious diseases and achieved good results [45, 46]. We can directly foresee future incidence trends through the ARIMA procedure and develop niche-targeting preventive and control measures. In addition, the 95% confidence interval range of the predictive value can also be used for early warning of infectious diseases. An infection during a specific period over the upper limit of the confidence interval may be a warning that a particular cluster or outbreak may occur. Although the forecasting result demonstrates a high degree of uncertainty, the overall trend indicates an upward. To date, no foresight studies utilizing the ARIMA model have been conducted regarding the prevalence of CL among IMP or the IGP across all provinces of Iran. However, several studies have focused on predictive analyses in specific provinces such as Esfahan and Fars. The results of these studies, similar to the current research, indicate a seasonal pattern of the disease. Nonetheless, the overall trend concerning the increase or decrease in the number of cases varies; some regions are experiencing an upward trend while others report a decline. This variability is expected, given that the vectors and reservoirs of Leishmania are influenced by climatic conditions, and there are significant climatic differences across various regions of Iran [47, 48]. In a study conducted by Charrahy et al., which investigated climate change and its impact on vulnerability to ZCL in Iran, the findings suggested that climate change is likely to favor an increase in the vectors and reservoirs of Leishmania. The study highlights that even in Ardabil, where CL is relatively low, future climatic changes could increase the disease risk. Additionally, climate changes are expected to raise the risk of CL in central, southern, and eastern Iran in the future [49].

Recent epidemiological data analysis indicates that Iran has made progress over the past years. However, the country still faces challenges in reducing the burden of CL. To ensure effective control programs within the healthcare system, it is crucial to enhance the country’s capacity by providing comprehensive training for staff and ensuring the availability of experienced clinical practitioners [50].

The present study encountered limitations regarding access to the health history of military personnel in Iran. The incidence of illness is likely higher than recorded statistics suggest; however, due to immunity generated from previous exposures, symptoms may not manifest during subsequent encounters. Consequently, these individuals may not be counted among the reported cases. Additionally, many patients did not complete the diagnostic process for various reasons. As a result, while the disease was diagnosed, the specific type of ACL or ZCL, was not determined.

## Conclusion

The unique conditions faced by military forces, especially frequent rotations in endemic areas, increase the risk of CL among newly deployed personnel who lack immunity. Our study emphasizes the impact of CL on IMP, particularly in central, south, southwestern, and western Iran. While overall cases have declined, provinces such as Esfahan exhibit an upward trend. The expansion of hotspots from the west and southwestern to the center and south of Iran, coupled with an upward trend in time series analysis, suggests the potential emergence of new foci and a rise in patient numbers in the future. To prevent disease transmission in endemic provinces, effective management of healthcare and control of disease vectors and reservoirs are critical. In provinces with high disease prevalence, these measures should be prioritized, particularly in Ilam, Khuzestan, and Esfahan.

## Supplementary Information

Supplementary Material 1.

Supplementary Material 2.

## Data Availability

“the dataset supporting the conclusion of this article is induced within the article.”

## Abbreviations

CL: Cutaneous leishmaniasis
WHO: World health organization
ZCL: Zoonotic cutaneous leishmaniasis
ACL: Anthroponotic cutaneous leishmaniasis
GIS: Geographical information system
IGP: Iran’s general population
IMP: Information military personnel
ARIMA: Autoregressive integrated moving average
SARIMA: Seasonal autoregressive integrated moving average
CI: Confidence interval
AR: Autoregressive
MA: Moving average
AIC: Akaike Information Criterion
BIC: Bayesian Information Criterion
SAR: Seasonal autoregressive coefficients
