# Supplementary material for: Analysis of cutaneous leishmaniasis among military personnel in the Islamic Republic of Iran: a spatiotemporal study between 2018 and 2022, trend forecasting based on ARIMA model
Source: BMC Infect Dis. 2024 Nov 16;24:1310. doi: 10.1186/s12879-024-10200-x (PMC11569613; doi:10.1186/s12879-024-10200-x)
Supplement: Supplementary file 1 — Supplementary Material 1. [file 12879_2024_10200_MOESM1_ESM.docx]

**Forecast with Best ARIMA Model for Frequency**

**Method**

| Seasonal period | 12 |
| --- | --- |
| Criterion for best model | Minimum AICc |
| Rows used | 60 |
| Rows unused | 0 |

**Model Selection**

| **Model (d = 0, D = 0)** | **LogLikelihood** | **AICc** | **AIC** | **BIC** |
| --- | --- | --- | --- | --- |
| **p = 2, q = 1, P = 3, Q = 0*** | **13319.8** | **-26623.4** | **-26625.6** | **-26610.9** |
| **p = 3, q = 2, P = 3, Q = 0** | **396.7** | **-768.8** | **-773.3** | **-752.4** |
| **p = 2, q = 2, P = 3, Q = 2** | **387.7** | **-751.0** | **-755.5** | **-734.5** |
| **p = 2, q = 0, P = 2, Q = 1** | **-136.8** | **289.8** | **287.6** | **302.3** |
| **p = 3, q = 0, P = 2, Q = 1** | **-142.4** | **303.7** | **300.9** | **317.6** |
| **p = 2, q = 0, P = 3, Q = 2** | **-218.8** | **459.1** | **455.5** | **474.4** |
| **p = 2, q = 2, P = 0, Q = 0** | **-260.0** | **533.5** | **532.0** | **544.5** |
| **p = 3, q = 2, P = 0, Q = 0** | **-259.9** | **536.0** | **533.9** | **548.5** |
| **p = 2, q = 0, P = 0, Q = 1** | **-263.3** | **537.8** | **536.7** | **547.1** |
| **p = 3, q = 1, P = 2, Q = 0** | **-259.9** | **538.7** | **535.9** | **552.6** |
| **p = 3, q = 0, P = 0, Q = 1** | **-263.2** | **540.0** | **538.4** | **551.0** |
| **p = 1, q = 0, P = 0, Q = 1** | **-266.7** | **542.1** | **541.4** | **549.7** |
| **p = 2, q = 0, P = 1, Q = 1** | **-265.4** | **544.5** | **542.9** | **555.5** |
| **p = 3, q = 0, P = 0, Q = 0** | **-266.8** | **544.8** | **543.7** | **554.2** |
| **p = 2, q = 0, P = 0, Q = 2** | **-266.4** | **546.5** | **544.9** | **557.4** |
| **p = 2, q = 0, P = 0, Q = 0** | **-269.0** | **546.7** | **546.0** | **554.4** |
| **p = 0, q = 1, P = 2, Q = 2** | **-266.1** | **548.3** | **546.2** | **560.8** |
| **p = 1, q = 1, P = 2, Q = 2** | **-264.9** | **548.7** | **545.9** | **562.6** |
| **p = 3, q = 0, P = 1, Q = 1** | **-266.4** | **549.0** | **546.9** | **561.6** |
| **p = 1, q = 0, P = 0, Q = 2** | **-269.1** | **549.2** | **548.1** | **558.6** |
| **p = 3, q = 0, P = 0, Q = 2** | **-266.5** | **549.2** | **547.1** | **561.7** |
| **p = 0, q = 2, P = 1, Q = 0** | **-269.6** | **550.3** | **549.2** | **559.7** |
| **p = 0, q = 2, P = 0, Q = 0** | **-271.8** | **552.4** | **551.7** | **560.0** |
| **p = 1, q = 1, P = 0, Q = 0** | **-271.9** | **552.5** | **551.8** | **560.2** |
| **p = 0, q = 1, P = 1, Q = 0** | **-272.2** | **553.1** | **552.3** | **560.7** |
| **p = 1, q = 1, P = 1, Q = 1** | **-270.5** | **554.5** | **552.9** | **565.5** |
| **p = 1, q = 1, P = 0, Q = 1** | **-272.1** | **555.3** | **554.2** | **564.7** |
| **p = 1, q = 0, P = 0, Q = 0** | **-275.7** | **557.8** | **557.4** | **563.7** |
| **p = 0, q = 0, P = 1, Q = 2** | **-274.6** | **560.4** | **559.3** | **569.7** |
| **p = 0, q = 1, P = 2, Q = 0** | **-275.1** | **561.4** | **560.3** | **570.7** |
| **p = 2, q = 1, P = 0, Q = 0** | **-275.4** | **561.9** | **560.8** | **571.2** |
| **p = 0, q = 2, P = 2, Q = 0** | **-274.3** | **562.2** | **560.6** | **573.2** |
| **p = 0, q = 1, P = 0, Q = 0** | **-278.9** | **564.2** | **563.8** | **570.1** |
| **p = 1, q = 2, P = 0, Q = 0** | **-277.2** | **565.5** | **564.4** | **574.9** |
| **p = 0, q = 1, P = 1, Q = 1** | **-277.3** | **565.7** | **564.6** | **575.1** |
| **p = 1, q = 1, P = 0, Q = 2** | **-276.5** | **566.6** | **565.0** | **577.5** |
| **p = 2, q = 2, P = 0, Q = 2** | **-274.2** | **567.1** | **564.3** | **581.1** |
| **p = 3, q = 2, P = 0, Q = 2** | **-273.2** | **568.1** | **564.5** | **583.3** |
| **p = 0, q = 0, P = 2, Q = 1** | **-278.8** | **568.6** | **567.5** | **578.0** |
| **p = 0, q = 2, P = 1, Q = 1** | **-277.7** | **569.0** | **567.4** | **580.0** |
| **p = 0, q = 0, P = 0, Q = 2** | **-280.9** | **570.6** | **569.8** | **578.2** |
| **p = 0, q = 0, P = 0, Q = 1** | **-282.2** | **570.8** | **570.4** | **576.7** |
| **p = 0, q = 2, P = 2, Q = 2** | **-276.1** | **571.1** | **568.3** | **585.0** |
| **p = 0, q = 2, P = 1, Q = 2** | **-277.9** | **572.0** | **569.9** | **584.6** |
| **p = 0, q = 1, P = 1, Q = 2** | **-279.4** | **572.4** | **570.8** | **583.4** |
| **p = 0, q = 0, P = 1, Q = 0** | **-284.4** | **575.3** | **574.9** | **581.2** |
| **p = 2, q = 2, P = 0, Q = 1** | **-280.5** | **577.2** | **575.1** | **589.7** |
| **p = 1, q = 2, P = 0, Q = 1** | **-281.9** | **577.4** | **575.8** | **588.3** |
| **p = 1, q = 2, P = 0, Q = 2** | **-281.2** | **578.6** | **576.4** | **591.1** |
| **p = 2, q = 0, P = 2, Q = 2** | **-280.1** | **579.0** | **576.1** | **592.9** |
| **p = 0, q = 0, P = 2, Q = 0** | **-286.0** | **580.8** | **580.1** | **588.5** |
| **p = 0, q = 1, P = 0, Q = 1** | **-286.5** | **581.8** | **581.1** | **589.5** |
| **p = 0, q = 2, P = 2, Q = 1** | **-283.1** | **582.3** | **580.1** | **594.8** |
| **p = 0, q = 2, P = 0, Q = 1** | **-286.4** | **583.8** | **582.7** | **593.2** |
| **p = 2, q = 2, P = 1, Q = 1** | **-282.5** | **583.9** | **581.1** | **597.8** |
| **p = 0, q = 1, P = 0, Q = 2** | **-287.4** | **585.9** | **584.7** | **595.2** |
| **p = 1, q = 2, P = 2, Q = 2** | **-282.6** | **586.9** | **583.3** | **602.1** |
| **p = 3, q = 2, P = 0, Q = 1** | **-284.5** | **587.8** | **585.0** | **601.7** |
| **p = 0, q = 2, P = 0, Q = 2** | **-287.8** | **589.3** | **587.7** | **600.2** |
| **p = 3, q = 2, P = 2, Q = 2** | **-282.3** | **592.1** | **586.6** | **609.6** |
| **p = 0, q = 0, P = 2, Q = 2** | **-289.5** | **592.5** | **590.9** | **603.5** |
| **p = 0, q = 1, P = 2, Q = 1** | **-289.8** | **593.2** | **591.6** | **604.2** |
| **p = 3, q = 1, P = 0, Q = 1** | **-290.8** | **597.7** | **595.5** | **610.2** |
| **p = 0, q = 0, P = 1, Q = 1** | **-295.8** | **598.0** | **597.5** | **603.8** |
| **p = 0, q = 1, P = 3, Q = 1** | **-295.3** | **606.7** | **604.5** | **619.2** |
| **p = 2, q = 1, P = 0, Q = 1** | **-298.7** | **610.9** | **609.3** | **621.9** |
| **p = 2, q = 1, P = 2, Q = 1** | **-296.1** | **611.1** | **608.2** | **625.0** |
| **p = 0, q = 2, P = 3, Q = 1** | **-297.0** | **612.8** | **609.9** | **626.7** |
| **p = 3, q = 1, P = 0, Q = 2** | **-298.1** | **615.0** | **612.1** | **628.9** |
| **p = 0, q = 1, P = 3, Q = 2** | **-298.9** | **616.7** | **613.9** | **630.6** |
| **p = 0, q = 0, P = 3, Q = 2** | **-302.6** | **621.3** | **619.2** | **633.8** |
| **p = 3, q = 2, P = 2, Q = 0** | **-300.2** | **622.0** | **618.4** | **637.3** |
| **p = 0, q = 1, P = 3, Q = 0** | **-305.9** | **622.8** | **621.7** | **632.2** |
| **p = 0, q = 2, P = 3, Q = 2** | **-301.6** | **624.9** | **621.3** | **640.1** |
| **p = 2, q = 1, P = 0, Q = 2** | **-304.8** | **625.8** | **623.7** | **638.4** |
| **p = 0, q = 0, P = 3, Q = 1** | **-307.8** | **626.8** | **625.6** | **636.1** |
| **p = 3, q = 2, P = 3, Q = 1** | **-301.8** | **631.2** | **625.7** | **648.7** |
| **p = 0, q = 0, P = 3, Q = 0** | **-312.0** | **632.8** | **632.1** | **640.4** |
| **p = 2, q = 1, P = 2, Q = 2** | **-307.4** | **636.4** | **632.8** | **651.6** |
| **p = 2, q = 2, P = 3, Q = 1** | **-313.2** | **650.8** | **646.3** | **667.3** |
| **p = 2, q = 2, P = 2, Q = 0** | **-318.3** | **655.4** | **652.5** | **669.3** |
| **p = 0, q = 2, P = 3, Q = 0** | **-323.5** | **660.5** | **658.9** | **671.5** |
| **p = 2, q = 2, P = 2, Q = 1** | **-321.3** | **664.3** | **660.7** | **679.5** |
| **p = 2, q = 2, P = 2, Q = 2** | **-325.3** | **675.1** | **670.7** | **691.6** |
| **p = 3, q = 1, P = 2, Q = 2** | **-325.7** | **676.0** | **671.5** | **692.4** |
| **p = 2, q = 1, P = 3, Q = 1** | **-478.2** | **975.2** | **972.4** | **989.1** |

** Best model with minimum AICc.  Output for the best model follows.*
