# Supplementary material for: Analysis of cutaneous leishmaniasis among military personnel in the Islamic Republic of Iran: a spatiotemporal study between 2018 and 2022, trend forecasting based on ARIMA model
Source: BMC Infect Dis. 2024 Nov 16;24:1310. doi: 10.1186/s12879-024-10200-x (PMC11569613; doi:10.1186/s12879-024-10200-x)
Supplement: Supplementary file 2 — Supplementary Material 2. [file 12879_2024_10200_MOESM2_ESM.docx]

**Forecasts from Time Period 60 ( 36 month after 2022)**

|  |  |  | **95% Limits** | |  |
| --- | --- | --- | --- | --- | --- |
| **Time Period** | **Forecast** | **SE Forecast** | **Lower** | **Upper** |  |
| 61 | 9.175 | 13.0061 | -16.322 | 34.672 |  |
| 62 | 22.052 | 14.1323 | -5.653 | 49.757 |  |
| 63 | 14.491 | 14.2981 | -13.539 | 42.521 |  |
| 64 | 18.486 | 14.3136 | -9.574 | 46.546 |  |
| 65 | 14.483 | 14.3136 | -13.577 | 42.543 |  |
| 66 | 38.745 | 14.3170 | 10.678 | 66.812 |  |
| 67 | 80.429 | 14.3239 | 52.348 | 108.509 |  |
| 68 | 97.069 | 14.3328 | 68.971 | 125.167 |  |
| 69 | 84.138 | 14.3426 | 56.021 | 112.255 |  |
| 70 | 80.851 | 14.3527 | 52.714 | 108.988 |  |
| 71 | 70.181 | 14.3630 | 42.024 | 98.338 |  |
| 72 | 19.800 | 14.3732 | -8.377 | 47.977 |  |
| 73 | 6.541 | 15.3549 | -23.561 | 36.643 |  |
| 74 | 17.605 | 15.4830 | -12.748 | 47.958 |  |
| 75 | 7.958 | 15.4898 | -22.408 | 38.324 |  |
| 76 | 13.961 | 15.4915 | -16.408 | 44.330 |  |
| 77 | 14.205 | 15.5007 | -16.182 | 44.593 |  |
| 78 | 31.979 | 15.5152 | 1.563 | 62.395 |  |
| 79 | 49.986 | 15.5324 | 19.536 | 80.435 |  |
| 80 | 75.236 | 15.5507 | 44.751 | 105.721 |  |
| 81 | 157.178 | 15.5694 | 126.656 | 187.700 |  |
| 82 | 96.361 | 15.5881 | 65.803 | 126.920 |  |
| 83 | 65.517 | 15.6068 | 34.922 | 96.113 |  |
| 84 | 43.203 | 15.6254 | 12.571 | 73.835 |  |
| 85 | 22.732 | 16.1508 | -8.930 | 54.394 |  |
| 86 | 12.777 | 16.2959 | -19.169 | 44.724 |  |
| 87 | 20.900 | 16.3482 | -11.149 | 52.949 |  |
| 88 | 12.760 | 16.3739 | -19.339 | 44.860 |  |
| 89 | 16.732 | 16.3907 | -15.400 | 48.865 |  |
| 90 | 59.687 | 16.4040 | 27.528 | 91.845 |  |
| 91 | 102.671 | 16.4158 | 70.490 | 134.853 |  |
| 92 | 109.479 | 16.4269 | 77.276 | 141.683 |  |
| 93 | 101.801 | 16.4376 | 69.577 | 134.025 |  |
| 94 | 43.457 | 16.4481 | 11.213 | 75.702 |  |
| 95 | 23.291 | 16.4585 | -8.974 | 55.556 |  |
| 96 | 28.483 | 16.4687 | -3.802 | 60.768 |  |
